# Prescribing Data in General Practice Demonstration (PDGPD) project - a cluster randomised controlled trial of a quality improvement intervention to achieve better prescribing for chronic heart failure and hypertension

**DOI:** 10.1186/1472-6963-12-273

**Published:** 2012-08-23

**Authors:** Margaret Williamson, Magnolia Cardona-Morrell, Jeffrey D Elliott, James F Reeve, Nigel P Stocks, Jon Emery, Judith M Mackson, Jane M Gunn

**Affiliations:** 1 Research & Development Team, National Prescribing Service, Level 7, 418a Elizabeth St, Surry Hills, NSW 2012, Australia; 2 Program Implementation Team, National Prescribing Service, Level 7, 418a Elizabeth St, Surry Hills, NSW 2012, Australia; 3 e-Health and Decision Support Team, National Prescribing Service, Level 6, 176 Wellington Parade, East Melbourne, VIC, 3002, Australia; 4 Discipline of General Practice, The University of Adelaide, 178 North Terrace, Adelaide, SA, 5005, Australia; 5 Department of General Practice, University of Western Australia, 35 Stirling Highway, Crawley, WA, 6009, Australia; 6 General Practice and Primary Health Care Academic Centre, The University of Melbourne, 200 Berkeley Street, Carlton, VIC, 3053, Australia

## Abstract

**Background:**

Research literature consistently documents that scientifically based therapeutic recommendations are not always followed in the hospital or in the primary care setting. Currently, there is evidence that some general practitioners in Australia are not prescribing appropriately for patients diagnosed with 1) hypertension (HT) and 2) chronic heart failure (CHF). The objectives of this study were to improve general practitioner’s drug treatment management of these patients through feedback on their own prescribing and small group discussions with peers and a trained group facilitator. The impact evaluation includes quantitative assessment of prescribing changes at 6, 9, 12 and 18 months after the intervention.

**Methods:**

A pragmatic multi site cluster RCT began recruiting practices in October 2009 to evaluate the effects of a multi-faceted quality improvement (QI) intervention on prescribing practice among Australian general practitioners (GP) in relation to patients with CHF and HT. General practices were recruited nationally through General Practice Networks across Australia. Participating practices were randomly allocated to one of three groups: two groups received the QI intervention (the prescribing indicator feedback reports and small group discussion) with each group undertaking the clinical topics (CHF and HT) in reverse order to the other. The third group was waitlisted to receive the intervention 6 months later and acted as a “control” for the other two groups.

De-identified data on practice, doctor and patient characteristics and their treatment for CHF and HT are extracted at six-monthly intervals before and after the intervention. Post-test comparisons will be conducted between the intervention and control arms using intention to treat analysis and models that account for clustering of practices in a Network and clustering of patients within practices and GPs.

**Discussion:**

This paper describes the study protocol for a project that will contribute to the development of acceptable and sustainable methods to promote QI activities within routine general practice, enhance prescribing practices and improve patient outcomes in the context of CHF and HT. Trial registration: Australian New Zealand Clinical Trials Registry (ANZCTR), Trial # 320870.

## Background

Pharmacological management of hypertension (HT) reduces cardiac events, stroke, hospitalisations, health care costs and improves quality of life for hypertensive patients (HT) [1,2]. Appropriate treatment of chronic heart failure (CHF) with ACE inhibitors, angiotensin II receptor antagonists, beta-blockers and diuretics have also shown benefits in terms of survival and averted hospitalisations, irrespective of the underlying cause [3,4].

Australian [5,6] and overseas studies report that treatment of HT [7-15] and heart failure [16-19] are not well aligned with evidence-based guidelines. For example, in Australia, fewer than 50 percent of heart failure patients admitted to any of three hospitals in Tasmania were being treated with target doses of the recommended drugs [18]. Among Australian patients attending general practice, under-prescribing for heart failure was found both in terms of the number receiving the recommended drugs and the dosage levels [20,21]. A national Australian survey reported the prevalence of untreated HT at 15.2% [13] and four consecutive GP audits of self-reported prescribing practices concluded that there was room for improvement in the management of hypertensive patients with co-morbidities [14].

The consequences of suboptimal care for these conditions include increased hospitalisation, higher mortality, [22,23] greater symptom severity [23] and increased costs to the health care system [22,24-26]. It is clear that best practice guidelines alone cannot secure improvements in practice [5,27-29].

Educational interventions and quality improvement (QI) activities can improve the quality of prescribing in general practice settings [5,30]. Multi-faceted interventions, particularly those involving interactive educational sessions for healthcare providers, and/or patient education are reported to be more effective than passive interventions [10,31-37]. Multi-faceted interventions have shown modest improvements in patient outcomes such as the proportion of patients meeting blood pressure targets [8,23] and also changes in prescribing patterns such as the proportion CHF patients receiving a beta blocker or taking target doses of ACE inhibitors [38].

In evaluating these types of complex QI interventions, pragmatic randomised controlled trials (RCTs) are emerging as a way of bridging the gap between traditional RCTs which have a good internal validity and observational studies, which have good external validity [39-41].

The Prescribing Data in General Practice Demonstration (PDGPD) project uses a multifaceted complex intervention aimed to improve GP prescribing behaviour for patients with HT and CHF in alignment with clinical practice guidelines.

This paper provides details of the intervention components and protocol, and methods and measurements used to evaluate the impact of the quality improvement initiative.

The project is a partnership between National Prescribing Service (also known as NPS MEDICINEWISE), an independent, evidence-based organisation delivering continuing education for health professionals to enable better decisions about medicines and medical tests [42], and the Australian General Practice Network (AGPN), the body representing a network of 111 local general practice networks (henceforth referred to as Networks) which covers 90% of registered GPs in the country [43].

The project governance included several external and internal groups that oversee various aspects of the project including the evaluation and the implementation. (see Additional file 1: Appendix 1).

## Methods/Design

### Aims of the intervention

The aim of the PDGPD intervention is to improve prescribing behaviour and clinical outcomes for patients with HT and CHF. These topics were chosen due to the existing gap between evidence and actual practice and the potential benefits resulting from changes in the management of patients. A list of terms used in clinical practice software to describe these two diagnoses can be found in Appendix 2 (Additional file 2). The quality improvement activity allows GPs to use their own prescribing data for ongoing review of patient management and for peer comparison.

### The intervention

The intervention included facilitated discussion about these conditions within general practices recruited across Australia using feedback about their patient management extracted from practice clinical software. This multi-faceted intervention was designed to be implemented from June 2009 with follow-up until December 2011.

**Figure 1:**
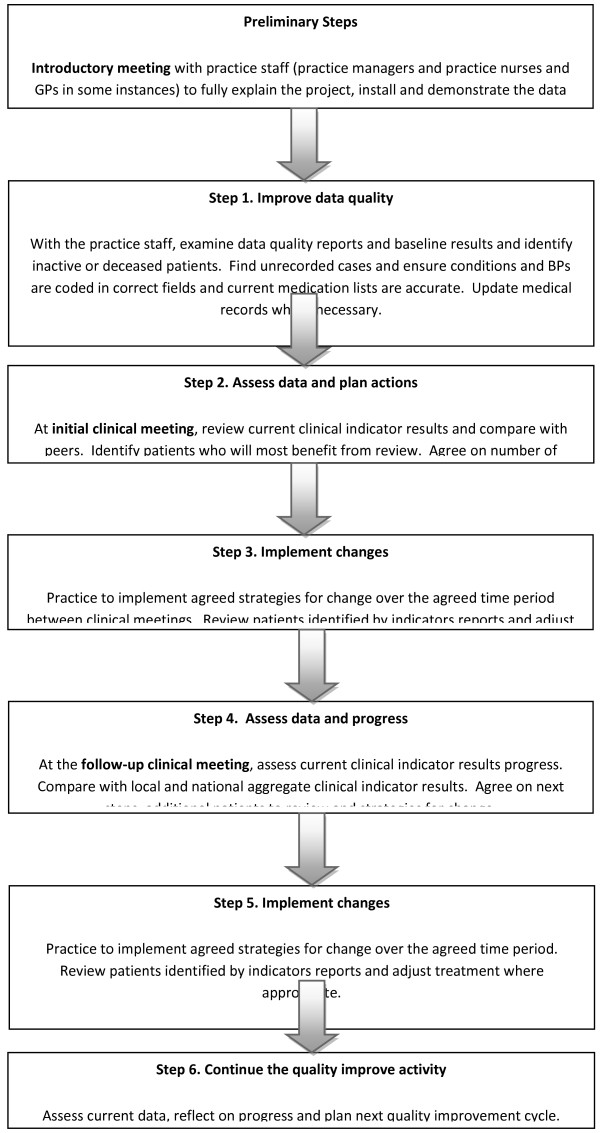
Quality improvement intervention process.

We used a multifaceted intervention based upon recognised quality improvement strategies that are implemented over a 12 month time frame. The intervention in this project consisted of a series of quality improvement activities (Figure 1). Data cleaning and recording of relevant clinical information was actively encouraged two months prior to the first clinical meeting among all practices in the study (step 1) to enable extraction of reliable information from relevant database fields. An initial facilitated group discussion (*clinical meeting*) was held by GPs within the practice with a trained project facilitator on treatment of CHF and HT (step 2). A data extraction and report tool supplied immediate feedback from the practice clinical software system regarding each GP’s own prescribing, as well as the results for the whole practice and the local GP network for peer comparisons for each clinical meeting.

Identification of patients suitable for clinical review occurred after the initial clinical meeting (step 3). A follow-up facilitated peer group clinical meeting was held around two to three months after the first meeting (step 4) to check progress on the patients prioritised for review of therapy and any changes to the prescribing data feedback.

### Aim of the evaluation

The aim of the evaluation of the PDGPD project was to estimate the impact of the quality improvement intervention on prescribing practice and to measure short term changes in blood pressure in patients with HT and CHF. The evaluation used a pragmatic randomised cluster controlled trial design.

### Study groups and randomisation

**Figure 2:**
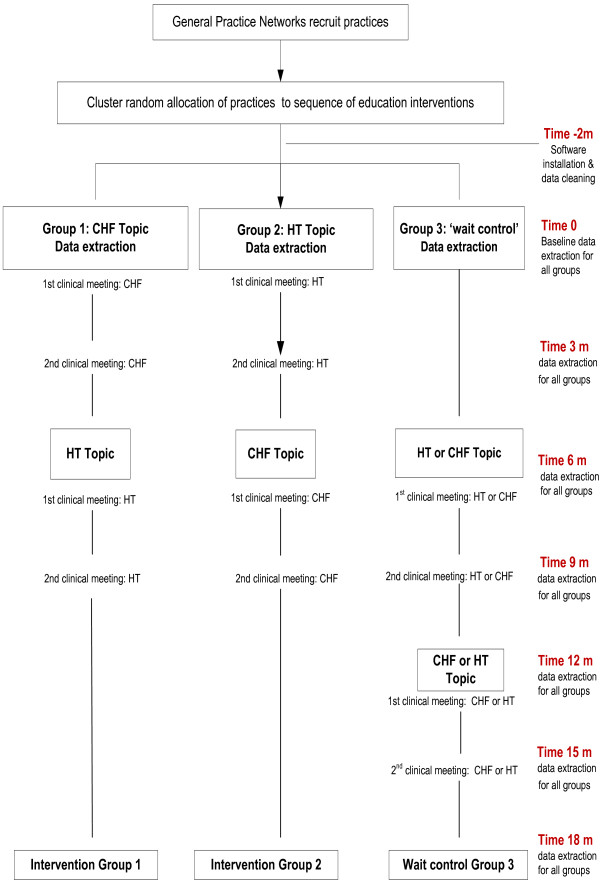
Evaluation using a cluster randomised controlled design. Group allocation and timeframes for data extraction and clinical meetings.

Practices agreeing to participate were registered and randomly allocated automatically into one of the three study groups (either into one of the two intervention groups or into the wait-control group) using computer generated random numbers. Block randomisation was used to enable balanced representation of practices in each of the Networks. The randomisation determined the sequence of exposure to the intervention as follows (see Figure 2):

Group 1 received the intervention for the CHF topic in the first six months and the HT topic in the next six months. In the first six months, this group also acts as the topic control group for group 2.

Group 2 received the intervention for the HT topic in the first six months and the CHF in the next six months and also acts as topic control group for group 1 in the first six months.

Group 3 is a wait-control for groups 1 and 2 in the first six months and did not receive any intervention in the first six months. This group received the intervention after six months and implemented the 2 topics sequentially – the order of topics was left up to the practice involved to choose. This group acts as ‘true’ control for groups 1 and 2 in the first six months.

As it is well recognised with educational interventions delivered at the practice level [40,44], it was not feasible to blind participating general practitioners and practices to the intervention because they had to be trained on the software installation and needed to be notified of their topic allocation in order to carry out the required quality improvement activity.

The expected time frame for the research was 2 years, including the recruitment and training time and 18 months of follow-up; data collection for baseline and six months are currently being analysed.

### Outcome assessment

Diagnoses of HT and CHF were based on GP clinical decision entered in their database. Clinical indicators of appropriate and safe prescribing behaviour and blood pressure control were used as measures of impact at various stages of the intervention. This type of indicator is widely used in the medical literature, and Australian doctors are familiar with the concept from their exposure to locally relevant educational resources and interventions extracting data from their medical software for other quality improvement initiatives [45-47].

The impact of the intervention was measured by two primary prescribing outcomes for each condition targeted by the intervention and five secondary prescribing outcomes relating to these two clinical topics. These were largely based on the prescribing indicators used in the feedback reports to the GPs. For details of the medicines covered see (Additional file 3: Appendix 3). Hospital admission for heart failure was not used as a clinical outcome indicator in this study because the prescribing software database does not link to the data collection for hospital admitted patients and does not contain coded fields for this information.

### Primary prescribing indicators

#### Hypertension

Proportion of adult patients with a diagnosis of HT who have suboptimal control of blood pressure as follows:

a. Proportion of adult patients with HT and using at least one antihypertensive drug, whose latest blood pressure is 140 / 90 mm Hg or higher

b. Proportion of adult patients with HT and coronary heart disease, diabetes, chronic kidney disease, stroke or transient ischaemic attacks (TIA), whose latest blood pressure is 130 / 80 mm Hg or higher

#### Chronic heart failure

Proportion of adult patients with CHF receiving appropriate treatment, ie:

a. using an ACE inhibitor or angiotensin II-receptor antagonist

b. using an ACE inhibitor or angiotensin II-receptor antagonist and using a heart-failure-specific beta blocker

### Secondary prescribing outcomes

#### Hypertension

1. Proportion of adult patients with HT using a prohypertensive drug whose latest blood pressure is 140 / 90 mm Hg or higher (see Additional file 3: Appendix 3)

2. Proportion of adult patients using an ACE inhibitor or angiotensin II-receptor antagonist, who are concurrently using a systemic nonsteroidal anti-inflammatory drug and a diuretic

3. Mean change in blood pressure at each interval for patients with and without co-morbidities (i.e. diabetes, CHD, chronic kidney disease, stroke or TIA)

#### Chronic Heart Failure

1. Proportion of adult patients with CHF using an ACE inhibitor below the recommended dose

2. Proportion of adult patients with CHF using a drug that may exacerbate the disease (see Additional file 3:Appendix 3)

The clinical indicators used in this study were developed in consultation with clinical experts, GPs, pharmacists, consumers and policy makers via focus groups.

The decision on the final indicators to be measured was made on the basis of their construct validity, face validity and operability or availability for data extraction from routine collections. Most of the suggested indicators were adopted from a previously published NPS manual [45] or NPS audits. A comprehensive review of the evidence relating to each indicator was conducted to establish construct validity. Refinement to ensure face validity was undertaken during a large workshop with GPs and other potential users. Using a simple questionnaire with a Likert scale, participants were asked to rate the accuracy of the indicators for measuring good prescribing practice, the usefulness for identifying patients receiving suboptimal treatment, and the usefulness for comparing their own prescribing practice with that of other GPs.

Final indicator definitions were decided by consensus using the above evidence during clinical reference group meetings, with input from expert Australian GPs and other advisory or steering groups where necessary.

Final selection of 9 indicators from a set of 82 potential clinical indicators was followed by field testing. The indicators were included in the study if there was evidence of their potential to improve patient care in the general practice setting, and they could be automatically extracted from general practice clinical software. Blood pressure measurements used in this study were those taken during the course of routine clinical care. All blood pressure measurements available in coded fields were retrieved at each interval but the latest available blood pressure recorded for each patient within 12 months of the data extraction was generally used to determine the latest blood pressure level; and up to 12 blood pressure readings per patient in the past year were used to estimate the mean for the practice.

### Data collection for the impact evaluation

The extraction tool enables de-identification of GP and patient data, encryption and transfer of information to a secure NPS file transfer site for the evaluation.

Data extracts to estimate indicators for evaluation by group allocation was planned to occur every three months after the initial clinical meeting.

### Sample Size estimations

Sample sizes were calculated with an assumed intra-cluster correlation of 0.08 at the 80% power and 5% significance level using the University of Aberdeen sample size calculator, (version 1.0.2) which adjusts for the clustering of GPs and patients in practices [48]. Estimates for the ICC and other parameters were based on previous studies and analysis of data from a sample of general practices [49]. With more than one primary outcome to be measured, the sample size required for the trial had to be powered to detect changes for the condition with lower prevalence. In Australian general practice, we applied the CHF clinical indicators as the basis of the sample size calculations (estimated prevalence: 1%-4.1% for CHF vs. 10%-44% for HT) [50-52]. Data previously collected from an Australian GP panel source,[49] were used for the following calculations. Assuming each of the GPs saw at least half of their patient clientele in a year, it was estimated that around 3% of GPs patient population had CHF and that there are 7200 active adult patients in an average 3 GP practice, of which 86 patients with CHF were expected to visit the practice during the first 6 months of the study. For a three-arm study with ICC = 0.08, the total number of practices required to show this difference is 99. Further, based on previous Australian and overseas experiences of GP or practice recruitment in trials and surveys [19,53,54], with an estimated drop out rate of 40-50% of all clusters (practices), at least 180 practices were required to identify both sufficient cases of CHF (the smaller patient group) and observable changes such as 10% absolute increase in prescribing of ACE inhibitors and 15% absolute increase in prescribing of beta blockers.

### Eligibility criteria for Networks, practices and GPs

To be eligible to participate, a Network had to agree to promote the study to their practices and GPs, be able to recruit a minimum of 7 and a maximum of 15 general practices, and have the capacity to appoint appropriate personnel (project facilitators and other relevant staff) to support the quality improvement intervention. For practices to be eligible, the practice principal GP had to consent to allow the installation of the data software extraction tool and consent for the data to be securely transferred for analysis. Consent was obtained from GPs participating in the PDGPD activities.

### Incentives for GPs, practices and Networks

GPs participating in two facilitated peer group clinical meetings and undertaking a review of their patients are eligible for mandatory continuing professional development points recognised by the Royal Australian College of General Practice and the Australian College of Rural and Remote Medicine. This quality prescribing initiative also makes the practice eligible for payment as part of the Australian Government’s Practice Incentives Program.

The funds provided to the Networks to deliver the project were deliberately weighted to ensure Networks recruited and retained the required minimum number of practices for the duration of the project. Networks were requested to provide $A500 to each practice to assist with data cleaning processes at the project start.

### Recruitment procedures

Recruitment of GP practices occurred in two stages. In stage 1, Networks were recruited through a call for Expressions of Interest from NPS and AGPN to recruit practices and deliver the intervention. In stage 2, Networks recruited practices and GPs to participate in the study and employed project facilitators to deliver and co-ordinate the intervention in participating practices in their Network.

### Project facilitator training

Two 2-day facilitator training workshops were delivered by NPS at the study start to give project facilitators a clear understanding of the Project, its aims, their roles and responsibilities and principles of research design, privacy issues and project implementation. Clinical knowledge of management of patients with HT and CHF was another important component. The focus then turned to developing skills in communication, negotiation and persuasion, group facilitation, problem solving, facilitating decision making, and change management. Data management skills covered demonstration of the software installation, data collection and cleaning, data transfer and storage and data interpretation as well as hands on use of the data extraction tool and practical examples on how to improve data quality. On-going monthly teleconferences with groups of facilitators were also established to support facilitators in the field.

### Data Extraction and data cleaning

A third party software tool was used to extract prescribing data from general practices. The tool was developed to: (1) provide *clinical indicator reports* to measure the clinical indicators; (2) provide *data quality reports* to identify missing or incorrect clinical data to ensure accurate calculation of clinical indicator reports; and (3) enable *data extracts* at baseline, and at regular intervals through the intervention, in order to evaluate changes in prescribing over the intervention period. The data extraction tool was developed over a period of 18 months and included functional specification development, software development, testing and piloting in general practice. The functional specifications included definitions for where data is stored in clinical software; how the data is coded or recorded, and the algorithms for calculating the clinical indicator.

A pre-intervention phase of two months included the installation of the data extraction software, training of practice staff in its use for data cleaning, upload and transfer (see Additional file 4:Appendix 4). Data quality reports obtained from the data software extraction tool allowed the practice to *clean* their data by improving the quality of coding and completeness of fields in the practice clinical software during these first two months.

Clinical indicator reports were collected from the GP clinical software just before the initial clinical meeting and every quarter. They were used within the practices to give individual GP and practice-level feedback. Evaluation data extracts retrieved anonymised data for patients with relevant conditions. This complete data subset with intervention-relevant variables extracted was transferred to a secure data repository located at NPS for the impact evaluation.

### Data Analysis

The unit of randomisation is the practice and unit of analysis is the patients accounting for clustering by practice (using *Proc surveyfreq* in SAS Enterprise Guide v4). The following analysis will be presented comparing the three groups:

a. Successive cross-sectional analyses at six-month intervals which include any practice with patient data available at each point. This will be to estimate point prevalences of each indicator before and after the intervention. Comparisons of practice proportions of patients meeting the individual guideline-recommended indicators (with 95% confidence intervals) across intervention groups will be conducted using χ^2^ statistics.

b. Longitudinal (cohort) estimates of changes in indicators occurring between time points, based on data from patients who had at least one visit before baseline and at least one visit following the intervention.

c. Sequential indicators will be calculated where changes in prescribing behaviours are measured only for patients not meeting guideline-driven indicators in the preceding 6-12-month period (e.g. proportion of CHF patients using an ACE inhibitor or angiotensin II-receptor antagonist at 6 months, out of those not receiving this treatment at baseline). This approach will show whether the intervention modified prescribing behaviour using a denominator where patients have scope for improvement.

Changes in the proportion of patients appropriately prescribed target medicines before and after the intervention will also be presented, with χ^2^ statistics for categorical measures (e.g. on heart failure medication or not at each time point), and significance testing of before and after the intervention effects within intervention groups based on paired t tests for continuous measures (e.g. mean blood pressure change) within groups and with a p-level of ≤0.01 considered to be statistically significant.

Supplementary regression analyses of the cohort will identify predictors of improved prescribing at 12 and 18 months after controlling for other possible confounders (determinants at the practice level) such as practice size, rural/urban location, socio-economic level of the area, percentage female doctors, mean years of GP experience. Post-test comparisons will be conducted between the intervention and wait-control arms using intention to treat analysis [55] and models that account for the non-independence of observations due to the clustering of practices in a Network and clustering of patients within practices and GPs. Separate binary logistic regression models will be analysed for CHF and HT adjusting for clustering effects of practice and GP [56]. Stepwise regression techniques will be used. Odds ratios will be used as the common measure of the estimated effect of the intervention and the independent effect of explanatory variables. To account for chance imbalance across intervention groups,[57] explanatory variables for each model will include patient characteristics, practice characteristics, and location of the Network. The researchers analysing the impact of the intervention on study outcomes have no contact with Networks or practices involved and are blinded to allocation group code at the baseline analysis stage.

### Ethical considerations

The study obtained ethics approvals from the Royal Australian College of General Practitioners Human Research Ethics Committee (approval number RACGP 08/007).

Participation of GPs and practices was voluntary and subject to informed consent for participation by the practice and the GP prior to any whole-of-practice data extraction. Although patients are not direct participants in the study, their non-identifiable data is being used to evaluate the intervention with GPs. Patients are notified via a poster in the practice that the practice is participating in a quality improvement initiative and can opt out of the process of their de-identified information being sent to NPS for analysis by notifying their doctor. After recording withdrawal of consent, their data are removed and not extracted by the data extraction tool used to collect the evaluation data.

Members of the research and evaluation team analysing the data have access only to de-identified information for Networks, practices and patients. Staff involved in data decryption do not participate in the analysis and all staff are required to sign a confidentiality agreement.

## Discussion

The challenges of implementing and evaluating real-world interventions to improve health and professional behaviours are well documented [27,30,58-60]. Participation of doctors in educational interventions and their willingness to accept evaluation audits rely on practice-based and doctor-based factors not often reported in the literature.[53,54] A doctor’s prescribing behaviour also depends on personal characteristics, years of experience, motivation, marketing strategies used by pharmaceutical industries, demands from society and patients, knowledge of guidelines, confidence and regulatory actions [34,60-63].

There are methodological difficulties in designing sound evaluations to identify the impact that context can have on success of prescribing interventions [54,57,64]. For instance, GPs prescribe in apparent conflict with guidelines for reasons that are complex, and can vary from previous experience with medical misadventures, to patient-factors such as patient age, education, ethnicity, social class, likely compliance and lifestyle considerations. [62,64] Uncertainty of intervention effectiveness is further compounded by the bias in reporting of studies and the inability to attribute success to individual intervention components [23,65].

The evaluation of PDGPD project uses a pragmatic cluster RCT design, where one of two interventions or control status is randomly assigned to individual practices. A pragmatic cluster-randomised trial is considered an appropriate research design in public health and educational interventions as it allows more variability in the entry criteria, reduces the impact of contamination within groups, and provides administrative convenience for implementation [39,40,57,66]. PDGPD will have access to several data points to investigate immediate and mid-term impact of the quality improvement initiative. However, several potential weaknesses in the design of PDGPD are acknowledged: first, the voluntary nature of GP participation in the cluster design where a practice [cluster] agreed to participate but not all GPs in the practice received the intervention. This may lead to dilution of the intervention effect when data from all patients seen by all GPs in the practice are analysed. The intervention is designed to encourage GPs to communicate key messages with other non-participating GPs in the practice and to welcome non-enrolled GPs to attend clinical discussion meetings within the same intervention group; further, the impact of participation rate by practice can be incorporated in the regression analysis model. Second, the cross-over design (Figure 1) where the same facilitator delivers one intervention after another in the same practice and one topic in one intervention practice and the *competing* topic in another practice carries the risk of cross-contamination; allocation of clinical topic order was done at random and facilitators were trained at focusing on discrete messages of one topic at a time. Thirdly, the wait-control group provides pre-intervention comparisons only for the first six months of the trial; for practical reasons in non-research settings (i.e. need for an intervention in exchange for participation) the control group received the intervention six months later.

Results of this evaluation will document the impact of an intervention to align GP prescribing behaviours with practice guidelines over time in Australia. Further, the evaluation will report the extent to which practice characteristics (such as rural/urban location and total number of active patients) and patient profile, (such as age, sex, presence of co-morbidities and number of medications) may affect the likelihood of adopting these prescribing changes. The ability to adopt or adapt evidence-based programs with fidelity in general practice is an important aspect to identify feasibility, acceptability and sustainability of such programs in the future. A comprehensive suite of qualitative studies have been developed for this project to assess such issues. Details on the qualitative *evaluation* components of the PDGPD will be described elsewhere.

Finally, the PDGPD project is expected to bring the following benefits:

• Improved care and outcomes for patients with heart failure and hypertension

• Acceptable and sustainable methods to promote quality improvement activities within general practice

• Purpose-built extraction and feedback tools that can calculate complex indicators useful to monitor prescribing behaviour, promote change and enable evaluation of educational interventions

• Opportunity to refine clinical indicators to better reflect quality of clinical care and outcomes of prescribing behaviour.

## Competing interests

The authors declare that they have no competing interests. Staff from the NPS Research and Development team had a role in project design and data analysis independently of the NPS health professional staff and decision support staff who designed the quality improvement activity, delivered the project facilitator training on the two clinical topics and managed the program implementation and data coordination. AGPN played an advisory role on the practicalities of working with, and in the recruitment of general practice networks, and had no involvement in data analysis or interpretation. The government funding body (Commonwealth Department of Health and Ageing) had no role in the conception or development of the study design, the writing of the manuscript or the decision to submit it for publication. This study has not received any funding from commercial organisations.

## Author’s contributions

MW conceived and developed the design of the study, developed ethics application and obtained ethics approval, conducted literature searches, undertook and supervised data quality assurance, led the first draft and revised this manuscript and commented on subsequent versions. MCM conducted literature searches, contributed to the definitions of prescribing indicators to measure impact, produced data specifications and analysis plan for the impact analysis plan and led subsequent drafts of the manuscript to completion. JDE contributed to indicator development, project design, project implementation, data coordination, and revision of the manuscript versions. JM provided input into project design, indicator development, clinical considerations and day-to-day strategic decisions on project direction. NS, JG, JR and JE made intellectual contributions into trial design and project implementation issues and commented on drafts of the manuscript. All authors read and approved the final manuscript.

## Pre-publication history

The pre-publication history for this paper can be accessed here:

http://www.biomedcentral.com/1472-6963/12/273/prepub

## Supplementary Material

Additional file 1 ** Appendix 1. **PDGPD Study Governance membership and functions. Click here for file

Additional file 2 **Appendix 2. **Scope of CHF and HT definitions. Click here for file

Additional file 3 ** Appendix 3. **Medicines relevant to the intervention. Click here for file

Additional file 4 ** Appendix 4. **Key Data Management and Data Cleaning Activities Undertaken by Facilitators or Practice Staff. Click here for file

## References

[B1] Chobanian AV Bakris GL Black HR Cushman WC Green LA Izzo JL Jr Jones DW Materson BJ Oparil S Wright JT Jr The seventh report of the joint national committee on prevention, detection, evaluation, and treatment of high blood pressure: the JNC 7 report JAMA 2003 289 19 2560 2572 10.1001/jama.289.19.2560 12748199

[B2] Guide to management of hypertension 2008; Assessing and managing raised blood pressure in adults - Updated December 2008 http://www.heartfoundation.org.au/SiteCollectionDocuments/HypertensionGuidelines2008to2010Update.pdf

[B3] Jhund PS Macintyre K Simpson CR Lewsey JD Stewart S Redpath A Chalmers JW Capewell S McMurray JJ Long-term trends in first hospitalization for heart failure and subsequent survival between 1986 and 2003: a population study of 5.1 million people Circulation 2009 119 4 515 523 10.1161/CIRCULATIONAHA.108.812172 19153268

[B4] Solomon SD Wang D Finn P Skali H Zornoff L McMurray JJV Swedberg K Yusuf S Granger CB Michelson EL Effect of candesartan on cause-specific mortality in heart failure patients. The Candesartan in Heart failure Assessment of Reduction in Mortality and morbidity (CHARM) Program. Circulation 2004 110 180 2183 10.1161/01.CIR.0000144474.65922.AA 15466644

[B5] Woodward MC Streeton CL Guttmann A Killer GT Peck RW Polypharmacy management among Australian veterans: improving prescribing through the Australian Department of Veterans' Affairs' prescriber feedback programme Internal Medicine 2008 38 95 100 10.1111/j.1445-5994.2007.01453.x 18005132

[B6] Senes S Penm E Australian Institute of Health and Welfare Medicines for cardiovascular health: are they used appropriately? Cardiovascular disease series No. 27 Cat CVD No. 36 2007 AIHW, Canberra

[B7] Holmes JS Shevrin M Goldman B Share D Blue Cross and Blue Shield of Michigan Translating research into practice: are physicians following evidence-based guidelines in the treatment of hypertension? Medical Care Research and Review 2004 61 4 453 473 10.1177/1077558704269501 15536209

[B8] Mitchell E Sullivan F Grimshaw JM Donnan PT Watt G Improving management of hypertension in general practice: a randmonised controlled trial of feedback derived from electronic patient data British Journal of General Practice 2005 55 94 101 15720929 PMC1463214

[B9] Nelson MR Reid CM Krum H McNeil JJ Factors influencing family physician adherence to hypertension treatment guideline recommendations on the initiation of pharmacotherapy: questionnaire survey American Journal of Cardiovascular Drugs 2003 3 6 437 441 10.2165/00129784-200303060-00006 14728063

[B10] Herbert CP Wright JM Maclure M Wakefield J Dormuth C Brett-MacLean P Legare J Premi J Better prescribing project: a randomized controlled trial of the impact of case-based educational modules and personal prescribing feedback on prescribing for hypertension in primary care Family Practice 2004 21 5 575 581 10.1093/fampra/cmh515 15367481

[B11] Varis J Savola H Vesalainen R Kantola I Treatment of hypertension in Finnish general practice seems unsatisfactory despite evidence-based guidelines Blood Pressure 2009 18 1–2 62 67 19353413 10.1080/08037050902840631

[B12] Bramlage P Thoenes M Kirch W Lenfant C Clinical practice and recent recommendations in hypertension management - reporting a gap in a global survey of 1259 primary care physicians in 17 countries Current Medical Research and Opinion 2007 23 4 783 791 10.1185/030079907X182077 17407635

[B13] Briganti EM Shaw JE Chadban SJ Zimmet PZ Welborn TA McNeil J Atkins RC Untreated hypertension among Australian adults: the 1999–2000 Australian Diabetes, Obesity and Lifestyle Study (AusDiab) Med J Aust 2003 179 135 139 12885281 10.5694/j.1326-5377.2003.tb05114.x

[B14] O'Riordan S Mackson J Weekes L Self-reported prescribing for hypertension in general practice Journal of Clinical Pharmacy & Therapeutics 2008 33 5 483 488 10.1111/j.1365-2710.2008.00939.x 18834362

[B15] Windak A Gryglewska B Tomasik T Narkiewicz K Yaphe J Grodzicki T Competence of Polish primary-care doctors in the pharmacological treatment of hypertension J Eval Clin Pract 2010 16 1 25 30 10.1111/j.1365-2753.2008.01107.x 20367812

[B16] Murphy NF Simpson CR McAlister FA Stewart S MacIntyre K Kirkpatrick M Chalmers J Redpath A Capewell S McMurray JJV National survey of the prevalence, incidence, primary care burden, and treatment of heart failure in Scotland Heart 2004 90 1129 1136 10.1136/hrt.2003.029553 15367505 PMC1768509

[B17] Majeed A Williams J de Lusignan S Chan T Management of heart failure in primary care after implementation of the National Service Framework for Coronary Heart Disease: a cross-sectional study Public Health 2005 119 2 105 111 10.1016/j.puhe.2004.06.006 15694957

[B18] Boyles PJ Peterson GM Blease MD Vial JH Undertreatment of congestive heart failure in an Australian setting J Clin Pharm Ther 2004 29 15 22 10.1046/j.1365-2710.2003.00531.x 14748893

[B19] Cleland JGF Cohen-Solal A Cosin Aguilar J Dietz R Eastaugh J Follath F Freemantle N Gavazzi A van Gilst WH Hobbs FDR Management of heart failure in primary care (the IMPROVEMENT of Heart Failure Programme): an international survey Lancet 2002 360 1631 1639 10.1016/S0140-6736(02)11601-1 12457785

[B20] Krum H Tonkin AM Currie R Djundjek R Johnston CI Chronic heart failure in Australian general practice. The Cardiac Awareness Survey and Evaluation (CASE) Study MJA 2001 174 439 444 11386587 10.5694/j.1326-5377.2001.tb143369.x

[B21] Clark RA Eckert KA Stewart S Phillips SM Yallop JJ Tonkin AM Krum H Rural and urban differentials in primary care management of chronic heart failure: new data from the CASE study Med J Aust 2007 186 441 445 17484704 10.5694/j.1326-5377.2007.tb00993.x

[B22] Rodgers JE Gattis Stough WG Underutilization of evidence-based therapies in heart failure: the pharmacist's role Supplement to Pharmacotherapy 2007 27 4 Pt 2 18S 28S 10.1592/phco.27.4part2.18S 17381371

[B23] Walsh JM McDonald KM Shojania KG Sundaram V Nayak S Lewis R Owens DK Goldstein MK Quality improvement strategies for hypertension management. A systematic review Medical Care 2006 44 7 646 657 10.1097/01.mlr.0000220260.30768.32 16799359

[B24] Nelson MR McNeil JJ Peeters A Reid CM Krum H PBS/RPBS cost implications of trends and guideline recommendations in the pharmacological management of hypertension in Australia, 1994–1998 Med J Aust 2001 174 11 565 568 11453328 10.5694/j.1326-5377.2001.tb143436.x

[B25] Elliot WJ The economic impact of hypertension J Clin Hypertens (Greenwich) 2003 5 3 Suppl 2 3 13 10.1111/j.1524-6175.2003.02463.x PMC8099256 12826765

[B26] Degli Esposti L Valpiani G Pharmacoeconomic burden of undertreating hypertension PharmacoEconomics 2004 22 14 907 928 10.2165/00019053-200422140-00002 15362928

[B27] Conti CR Cooper-DeHoff RM How will INVEST and other hypertension trials change clinical practice? Clin Cardiol 2001 24 11 (Suppl) V242 229 10.1002/clc.4960241708 PMC6655238 11712773

[B28] van der Wel M Bakx C de Grauw W van Gerwen W Mulder J van Weel C The influence of guideline revisions on the process and outcome of hypertension management in general practice: A descriptive study European Journal of General Practice 2008 14 Suppl 1 47 52 18949645 10.1080/13814780802436200

[B29] Hetlevik I Holmen J Kruger O Kristensen P Iversen H Implementing Clinical Guidelines in the Treatment of Hypertension in General Practice Blood Pressure 1998 7 5–6 270 276 10321438 10.1080/080370598437114

[B30] Horn FE Mandryk JA Mackson JM Wutzke SE Weekes LM Hyndman RJ Measurement of changes in antihypertensive drug utilisation following primary care educational interventions Pharmacoepidemiol Drug Saf 2007 16 3 297 308 10.1002/pds.1243 16634120

[B31] Fretheim A Oxman A Havelsrud K Treweek S Kristoffersen DT Bjorndal A Rational Prescribing in Primary Care (RaPP): a cluster randomized trial of a tailored intervention PLoS Medicine 2006 3 6 783 791 10.1371/journal.pmed.0030134 PMC1472695 16737346

[B32] O'Connell DL Henry D Tomlins R Randomised controlled trial of effect of feedback on general practioners' prescribing in Australia Bmj 1999 318 507 511 10.1136/bmj.318.7182.507 10024260 PMC27749

[B33] Roumie CL Elasy TA Greevy R Griffin MR Liu X Stone WJ Wallston KA Dittus RS Alvarez V Cobb J Improving blood pressure control through provider education, provider alerts, and patient education Ann Intern Med 2006 145 165 175 16880458 10.7326/0003-4819-145-3-200608010-00004

[B34] Farmer AP Légaré F Turcot L Grimshaw J Harvey E McGowan JL Wolf F Printed educational materials: effects on professional practice and health care outcomes Cochrane Database of Systematic Reviews 2009 Issue 3 Art No: CD004398101002/14651858CD004398pub2 10.1002/14651858.CD004398.pub2 18646106

[B35] Forsetlund L Bjørndal A Rashidian A Jamtvedt G O'Brien MA Wolf F Davis D Odgaard-Jensen J Oxman AD Continuing education meetings and workshops: effects on professional practice and health care outcomes Cochrane Database Syst Rev 2009 15 CD003030 10.1002/14651858.CD003030.pub2 PMC7138253 19370580

[B36] Goldstein M Lavori P Coleman R Advani A Hoffman B Improving adherence to guidelines for hypertension drug prescribing: cluster-randomized controlled trial of general versus patient-specific recommendations Am J Managed Care 2005 11 677 685 16268751

[B37] Wahl C Gregoire JP Teo K Beaulieu M Labelle S Leduc B Cochrane B Lapointe L Montague T Concordance, compliance and adherence in healthcare: closing gaps and improving outcomes Healthcare Quarterly 2005 8 1 65 70 15715337 10.12927/hcq..16941

[B38] Hickey A Scott I Denaro C Using clinical indicators in a quality improvement programme targeting cardiac care International Journal of Quality in Health Care 2004 16 suppl 1 i11 i25 10.1093/intqhc/mzh032 15059983

[B39] Hotopf M The pragmatic randomised controlled trial Adv Psychiatr Treat 2002 8 5 326 333 10.1192/apt.8.5.326

[B40] Godwin M Ruhland L Casson I MacDonald S Delva D Birtwhistle R Lam M Seguin R Pragmatic controlled clinical trials in primary care: the struggle between external and internal validity BMC Medical Research Methodology 2003 3 28 http://www.biomedcentral.com/1471-2288/1473/1428 10.1186/1471-2288-3-28 14690550 PMC317298

[B41] Campbell MK Mollison J Steen N Grimshaw JM Eccles M Analysis of cluster randomized trials in primary care: a practical approach Family Practice 2000 17 2 192 196 10.1093/fampra/17.2.192 10758085

[B42] NPS for a medicinewise Australia- about ushttp://www.nps.org.au/about_us

[B43] Australian General Practice Network - about ushttp://www.agpn.com.au/about-us

[B44] Moore H Summerbell CD Vail A Greenwood DC Adamson AJ The design features and practicalities of conducting a pragmatic cluster randomized trial of obesity management in primary care Statistics in Medicine 2001 20 3 331 340 10.1002/1097-0258(20010215)20:3<331::AID-SIM795>3.0.CO;2-K 11180304

[B45] Indicators of Quality Prescribing in Australian General Practice. A manual for usershttp://www.nps.org.au/__data/assets/pdf_file/0019/37351/indicators_full.pdf

[B46] Voorham J Denig P Wolffenbuttel BHR Haaijer-Ruskamp FM Cross-sectional versus sequential quality indicators of risk factor management in patients with type 2 Diabetes Medical Care 2008 46 2 133–141 110.1097/MLR.1090b1013e31815b31819da31810 10.1097/MLR.0b013e31815b9da0 18219241

[B47] The APCC Program http://www.apcc.org.au/about_the_APCC/the_collaborative_program/

[B48] Health Services Research Unit University of Aberdeen Sample size calculation for cluster randomized trials in healthhttp://www.abdn.ac.uk/hsru/uploads/files/calculationmanual.pdf

[B49] General Practice Research Network (GPRN) Database description http://www.pbs.gov.au/info/industry/useful-resources/sources/GPRN

[B50] AIHW Australian GP Statistics and Classification Centre SAND abstract No. 90 from the BEACH program: Prevalence, management and investigations of chronic heart failure in general practice patients 2006 AGPSCC University of Sydney, Sydney

[B51] Britt H Miller GC Charles J Henderson J Bayram C Harrison C Valenti L Fahridin S Pan Y O’Halloran J General practice activity in Australia 2007–08. Cat. no. GEP 22 General practice series no 22 2008 Australian Institute of Health and Welfare, Canberra

[B52] Sturm JW Davis SM O'Sullivan JG Vedadhaghi ME Donnan GA The Avoid Stroke as Soon as Possible (ASAP) general practice stroke audit Medical Journal of Australia 2002 176 7 312 316 12013322 10.5694/j.1326-5377.2002.tb04430.x

[B53] Williamson M Pirkis J Pfaff J Tyson O Sim Kerse N Lautenschlager N Stocks N Almeida O Recruiting and retaining GPs and patients in intervention studies: the DEPS-GP project as a case study BMC Medical Research Methodology 2007 7 1 1 9 10.1186/1471-2288-7-1 17875219 PMC2147023

[B54] McKay-Brown L Borland R Balmford J Segan CJ Andrews C Tasker C Piterman L The challenges of recruiting and retaining GPs in Research: findings from a smoking cessation project Australian Journal of Primary Health 2007 13 61 67

[B55] Mugglin AS Connett JE D'Agostino RMJ, Sullivan L Analysis Population Wiley Encyclopedia of Clinical Trials 2008 John Wiley & Sons, Inc 1 8

[B56] Christie J O'Halloran P Stevenson M Planning a cluster randomized controlled trial - Methodological Issues Nursing Research 2009 58 2 128 134 10.1097/NNR.0b013e3181900cb5 19289934

[B57] Klar N Donner A Current and future challenges in the design and analysis of cluster randomization trials Statistics in Medicine 2001 20 3729 3740 10.1002/sim.1115 11782029

[B58] Kasje WN Denig P Stewart RE De Graeff PA Haaijer-Ruskamp FM An educational programme for peer review groups to improve treatment of chronic heart failure and diabetes mellitus type 2 in general practice J Eval Clin Pract 2006 12 6 613 621 10.1111/j.1365-2753.2005.00625.x 17100860

[B59] Phillips SM Tofler RLMGH Barriers to diagnosing and managing heart failure in primary caremedical Journal of Australia 2004 181 78 81 15257642 10.5694/j.1326-5377.2004.tb06178.x

[B60] Fuat A Hungin APS Murphy JJ Barriers to accurate diagnosis and effective management of heart failure in primary care: qualitative study Bmj 2003 326 7382 196 10.1136/bmj.326.7382.196 12543836 PMC140276

[B61] Nilsson G Hjemdahl P Hassler A Vitols S Wallen NH Krakau I Feedback on prescribing rate combined with problem-oriented pharmacotherapy education as a model to improve prescribing behavior among general practioners Eur J Clin Pharmacol 2001 56 843 848 10.1007/s002280000242 11294376

[B62] Bradley CP Factors which influence the decision whether or not to prescribe: the dilemma facing general practitioners British Journal of General Practice 1992 42 454 458 1472390 PMC1372266

[B63] Sinha S Schwartz MD Qin A Ross JS Self-Reported and Actual Beta-Blocker Prescribing for Heart Failure Patients: Physician Predictors PLoS One 2009 4 12 e8522 10.1371/journal.pone.0008522 20046824 PMC2796176

[B64] Freeman AC Sweeney K Why general practioners do not implement evidence: qualitative study Bmj 2001 323 1 5 10.1136/bmj.323.7303.1 11701576 PMC59686

[B65] Lindenauer PK Effects of quality improvement collaboratives Bmj 2008 336 7659 1448 1449 10.1136/bmj.a216 18577558 PMC2440851

[B66] Murray DM Varnell SP Blitstein JL Design and Analysis of Group-Randomized Trials: A Review of Recent Methodological Developments Am J Public Health 2004 94 3 423 432 10.2105/AJPH.94.3.423 14998806 PMC1448268

