# Supplementary material for: Prescribing Data in General Practice Demonstration (PDGPD) project - a cluster randomised controlled trial of a quality improvement intervention to achieve better prescribing for chronic heart failure and hypertension
Source: BMC Health Serv Res. 2012 Aug 23;12:273. doi: 10.1186/1472-6963-12-273 (PMC3515472; doi:10.1186/1472-6963-12-273)
Supplement: Additional file 1 — Appendix 1. PDGPD Study Governance membership and functions. [file 1472-6963-12-273-S1.docx]

Appendix 1: PDGPD Study Governance membership and functions

The overall structure of the governance of the PDGPD Project illustrated below consisted of several groups with complementary functions.

**The PDGPD External Steering Committee** provided the overall strategic direction for the PDGPD Project and AGPN as our collaborating partner was represented on this group, as well as representation from the Networks and GPs with an interest in this area.

**The NPS Internal Steering Committee** (formerly known as the Internal Governance Group) consisted of the Project Sponsor, project manager and managers from the Health Professional, Decision Support, R&D, Data Management and IT teams. The role of the Group was to provide overall management and corporate accountability for the project, ensuring proper governance.

**The Project Implementation Group** consisted of members from AGPN and NPS and was used as a forum to discuss stakeholder issues, including selection of Networks for the expressions of interest process, discussion and agreement of the roles and responsibilities of both parties, formulation of a communications plan and managing Network and practice relationships.

**The Clinical Reference Group** consisted of GPs with a knowledge of Australian Primary Care Collaboratives methodology and experience of extractable clinical indicators and whose function was to provide clinical knowledge, feedback on the definition, suitability and usability of the data extraction software and input into the intervention design.

**The Study Guidance Group’s** main function was to provide advice and direction for the development, conduct, analysis and reporting of evaluation of the project.

**Software Governance Group** was an internal group providing direction and final decisions for the development and testing of data extraction software tools.
