# Supplementary material for: Prescribing Data in General Practice Demonstration (PDGPD) project - a cluster randomised controlled trial of a quality improvement intervention to achieve better prescribing for chronic heart failure and hypertension
Source: BMC Health Serv Res. 2012 Aug 23;12:273. doi: 10.1186/1472-6963-12-273 (PMC3515472; doi:10.1186/1472-6963-12-273)
Supplement: Additional file 2 — Appendix 2. Scope of CHF and HT definitions. [file 1472-6963-12-273-S2.docx]

**Appendix 2: Scope of CHF and HT definitions**

| **Include patients:** | **Terms used in the indicator specifications to describe hypertension in the clinical software** | |
| --- | --- | --- |
| Hypertension | Essential hypertension  HBP  High blood pressure  HT (Hypertension)  Hypertension  Hypertension - Controlled  Hypertension - Isolated Systolic  Hypertension - Labile  Hypertension - Malignant  Hypertension - Renovascular  Hypertension – Unstable  Labile Hypertension  Malignant hypertension  Primary hypertension  Renal Hypertension  Reno-vascular Hypertension  Severe refractory hypertension  Blood Pressure Labile  BP Labile  BP Unstable  Labile BP  Labile Blood Pressure | **Excludes**  Possible hypertension  White coat HT  HT-pregnancy  HT-borderline  HT-stress related  HT investigation  HT-one reading  Anxiety provoked HT  HT- lifestyle management  HT-preventive care  Hypertensive heart failure  ? hypertension |

| **Include patients:** | **Terms used in the indicator specifications to describe chronic heart failure** | |
| --- | --- | --- |
| Chronic heart failure or  left ventricular dysfunction | Cardiac failure  CCF  Congestive Cardiac Failure  Congestive Heart Failure  Heart failure - Biventricular  Heart failure - High output  Heart failure - Left  Heart failure - Right  High output cardiac failure  High output heart failure Hypertensive heart failure  Left Ventricular Failure  LHF (Left Heart Failure)  LVF (Left ventricular failure)  Pulmonary oedema  RHF (Right heart failure)  Right ventricular failure  RVF (Right ventricular failure)  Left Ventricular Dysfunction  Ventricular dysfunction – left  Poor left ventricular function  Cor pulmonale | **Excludes**  Acute heart failure  Diastolic heart failure  Possible CHF  ? heart failure  CHF investigation |
