# Supplementary material for: Prescribing Data in General Practice Demonstration (PDGPD) project - a cluster randomised controlled trial of a quality improvement intervention to achieve better prescribing for chronic heart failure and hypertension
Source: BMC Health Serv Res. 2012 Aug 23;12:273. doi: 10.1186/1472-6963-12-273 (PMC3515472; doi:10.1186/1472-6963-12-273)
Supplement: Additional file 3 — Appendix 3. Medicines relevant to the intervention. [file 1472-6963-12-273-S3.docx]

**Appendix 3: Medicines relevant to the intervention**

**ANTI-HYPERTENSIVES**

| **Centrally-acting antihypertensives (A)** | Clonidine  Methyldopa  Moxonidine |  |
| --- | --- | --- |
| **ACE inhibitors (B)** | Captopril  Enalapril or enalapril maleate  Fosinopril or fosinopril sodium  Lisinopril  Perindopril or perindopril arginine or perindopril erbumine  Quinapril or quinapril hydrochloride  Ramipril  Trandolapril |  |
| **Thiazide or thiazide-like Diuretics (C)** | Hydrochlorothiazide  Chlorthalidone  Indapamide |  |
| **ACE inhibitor plus thiazide**  **or thiazide-like diuretic (B+C)** | Enalapril/hydrochlorothiazide  Fosinopril/hydrochlorothiazide  Perindopril/indapamide  Quinapril/hydrochlorothiazide |  |
| **Calcium-channel blockers (D)** | Amlodipine or amlodipine besylate or amlodipine maleate  Felodipine  Lercanidipine or lercanidipine hydrochloride  Nifedipine  Diltiazem (controlled delivery) or diltiazem hydrochloride (controlled delivery)  Verapamil or verapamil hydrochloride |  |
| **ACE inhibitor plus calcium channel blocker (B+D)** | Enalapril/lercanidipine hydrochloride  Ramipril/felodipine  Trandolapril/verapamil or trandolapril/verapamil hydrochloride  Perindopril/Amlodipine |  |
| **Angiotensin II-receptor**  **antagonists (E)** | Candesartan or candesartan cilexetil  Eprosartan or eprosartan mesylate  Irbesartan  Losartan or losartan potassium  Olmesartan or olmesartan medoxomil  Telmisartan  Valsartan |  |
| **Calcium channel blocker plus statin (D)** | Amlodipine/atorvastatin or amlodipine besylate/atorvastatin calcium |  |
| **Angiotensin II-receptor antagonist**  **plus thiazide diuretic (E+C)** | Candesartan/hydrochlorothiazide or candesartan cilexetil/hydrochlorothiazide  Eprosartan/hydrochlorothiazide or eprosartan mesylate/hydrochlorothiazide  Irbesartan/hydrochlorothiazide  Olmesartan/hydrochlorothiazide or olmesartan medoxomil/hydrocholorothiazide  Telmisartan/hydrochlorothiazide  Valsartan/hydrochlorothiazide |  |
|  |  |  |
| **Angiotensin II-receptor antagonist plus calcium channel blocker (E+D)** | Amlodipine/valsartan or amlodipine besylate/valsartan  Amlodipine/olmesartan |  |
|  |  |  |
| **Beta blockers (F)** | Atenolol  Bisoprolol or bisoprolol fumarate  Carvedilol  Esmolol  Labetalol or labetolol hydrochloride  Metoprolol or metoprolol succinate or metoprolol tartrate  Oxprenolol or oxprenolol hydrochloride  Pindolol  Propranolol or propranolol hydrochloride |  |
| **Potassium-sparing diuretic (G)** | Amiloride  Triamterene |  |
| **Thiazide plus potassium-sparing diuretic (C+G)** | Hydrochlorothiazide/amiloride  Hydrochlorothiazide/triamterene |  |
| **Alpha-blockers (selective) (H)** | Prazosin or prazosin hydrochloride  Terazosin or terazosin hydrochloride |  |
| **Vasodilators (I)** | Hydralazine or hydralazine hydrochloride  Minoxidil |  |
| **PRO-HYPERTENSIVES** |  |  |
| **Corticosteroid-Glucocorticoid.**  **Corticosteroid-Mineralocorticoid.** | Corticosteroid – Glucocorticoid class – include all  Corticosteroid – Mineralocorticoid class – include all |  |
| **Hormone replacement therapy (HRT) and oral contraceptives** | Hormone Replacement Therapy (HRT) class – include all  Hormone Replacement Therapy (HRT) Oestrogen only class – exclude topical oestrogens  Oestrogen/Progestogen Combinations as OCs class – include all  Progestogen/Oestrogen Combinations as OCs class – include all  Oral Contraceptives – Combined class – include all  Contraceptive - Oral Combined class – include all  Oestrogenic/Progestogenic Combinations as HRT class – include all  Progestogenic/Oestrogenic Combinations as HRT class – include all |  |
| **Systemic Nonsteroidal anti-inflammatory agents (NSAIDs)** | 'NSAIDs Systemic', 'Nonsteroidal anti-inflammatory agents Systemic', 'Systemic NSAIDs' including:  Diclofenac or diclofenac sodium or diclofenac potassium  Ibuprofen or ibuprofen lysine  Ibuprofen/pseudoephedrine hydrochloride  Indomethacin  Ketoprofen  Ketorolac or ketorolac trometamol  Mefenamic acid  Naproxen or naproxen sodium  Piroxicam  Sulindac  Tiaprofenic acid  Diclofenac sodium/misoprostol  Ibuprofen/codeine phosphate  Do not include: glucosamine, glucosamine sulfate, glucosamine hydrochloride, glucosamine hydrochloride/chondroitin sulfate, glucosamine sulfate/potassium chloride, chondroitin, chondroitin sulphate, methylsulfonylmethane/glucosamine hydrochloride/chondroitin sulfate sodium/ascorbic acid/citrus bioflavonoids/zinc/manganese/copper/boron, triclosanthes kirilowii/prunella vulgaris/clematis sinensis – exclude topical NSAIDs |  |
| **Anti-androgen and other hormones** | Cyproterone Acetate/Ethinyloestradiol  Dienogest/Oestradiol Valerate  Nomegestrol Acetate/Oestradiol |  |
| **Cyclooxygenase 2 COX 2) inhibitors** | Include all  Celecoxib  Meloxicam  Parecoxib or parecoxib sodium  Etoricoxib |  |
| **MAO inhibitors** | Include all  Phenelzine  Tranylcypromine |  |
| **Decongestant – Systemic** | Include drugs with ephedrine and pseudoephedrine and/or phenylephrine (alone or in combination products) |  |
| [**Sympathomimetics**](http://www.mims.co.uk/search/Drugs/phrase/Sympathomimetics/) | Adrenaline or adrenaline hydrochloride, Noradrenaline |  |
| **Generic drug names (no applicable classes available)** | Bromocriptine, bromocriptine (as mesylate), clozapine,  cyclosporine, darbepoetin, darbepoetin alfa, epoetin, epoetin alfa, epoetin beta, leflunomide, moclobemide, nicotine, sibutramine, sibutramine hydrochloride, phentermine, tacrolimus, reboxetine, reboxetine mesilate, venlafaxine, venlafaxine hydrochloride,  dehydro-epi-androsteron (DHEA), melatonin, hypericum,  hypericum perforatum, hypericum perforatum standardised,  St. John's wort, hypericum perforatum/L-tyrosine/L-glutamine/magnesium, hypericum perforatum (St. John's wort) dry flower herb top, hypericum perforatum/passion flower/gotu kola/gingko biloba/green tea, hypericum/eleutherococcus/vitamins. | |
| **DRUGS THAT EXACERBATE CHF** | **MD drug generic name or brand**  **name in ‘ ’** |  |
|  |  |  |
| **Antiarrhythmic class I**  **Antiarrhythmic class III** | **Antiarrhythmic class I – include all**  Disopyramide  Flecainide or flecainide acetate  **Antiarrhythmic class III – include only**  Sotalol or sotalol hydrochloride |  |
|  |  |  |
| **Non dihydropyridine calcium**  **channel blockers**  **(These two drugs are a subset of**  **The calcium channel blocker class)** | Verapamil or verapamil hydrochloride  Diltiazem or diltiazem hydrochloride or diltiazem hydrochloride (controlled delivery) |  |
| **Tricyclic Antidepressants** | Amitriptyline or amitriptyline hydrochloride  Clomipramine or clomipramine hydrochloride  Dothiepin or dothiepin hydrochloride  Doxepin or doxepin hydrochloride  Imipramine or imipramine hydrochloride  Nortiptyline or nortiptyline hydrochloride  Trimipramine or trimipramine maleate |  |
| **Systemic Nonsteroidal anti-inflammatory agents (NSAIDs)** | **Include the following from the Systemic NSAIDs class**  Diclofenac or diclofenac sodium or diclofenac potassium  Ibuprofen or ibuprofen lysine  Ibuprofen/pseudoephedrine hydrochloride  Indomethacin  Ketoprofen  Ketorolac or ketorolac trometamol  Mefenamic acid  Naproxen or naproxen sodium  Piroxicam  Sulindac  Tiaprofenic acid  Diclofenac sodium/misoprostol  Ibuprofen/codeine phosphate  **Do not include:**  Glucosamine or glucosamine sulphate or glucosamine hydrochloride  Glucosamine hydrochloride/chondroitin sulfate  Glucosamine sulfate/potassium chloride  Chondroitin or chondroitin sulphate  Methylsulfonylmethane/glucosamine hydrochloride/chondroitin sulfate sodium/ascorbic acid/citrus bioflavonoids/zinc/manganese/copper/boron  Triclosanthes kirilowii/prunella vulgaris/clematis sinensis |  |
| **COX 2 inhibitors** | **Includes all from Cyclooxygenase 2 inhibitors class**  Celecoxib  Meloxicam  Parecoxib or parecoxib sodium  Etoricoxib |  |
| **Glitazones**  **(Drugs from the antidiabetic**  **agents class)** | Pioglitazone or pioglitazone hydrochloride  Rosiglitazone  Rosiglitazone/metformin |  |
| **Corticosteroid-Glucocorticoid class** | Betamethasone or betamethasone acetate or betamethasone sodium phosphate  Budesonide (oral capsules only – brand name ‘Entocort’)  Cortisone or cortisone acetate  Dexamethasone  Fludrocortisone or fludrocortisones acetate  Hydrocortisone or hydrocortisone acetate or hydrocortisone sodium succinate  Methylprednisolone or methylprednisolone acetate or methylprednisolone sodium succinate  Prednisolone or prednisolone sodium phosphate  Prednisone  Triamcinolone or triamcinolone acetonide |  |
| **Tumour Necrosis Factor Receptor Blockers** | Adalimumab  Etanercept  Infliximab |  |
