# Supplementary material for: Prescribing Data in General Practice Demonstration (PDGPD) project - a cluster randomised controlled trial of a quality improvement intervention to achieve better prescribing for chronic heart failure and hypertension
Source: BMC Health Serv Res. 2012 Aug 23;12:273. doi: 10.1186/1472-6963-12-273 (PMC3515472; doi:10.1186/1472-6963-12-273)
Supplement: Additional file 4 — Appendix 4. Key Data Management and Data Cleaning Activities Undertaken by Facilitators or Practice Staff. [file 1472-6963-12-273-S4.docx]

Appendix 4: Key Data Management and Data Cleaning Activities Undertaken by Facilitators or Practice Staff

- Archive deceased patients and those who have not been in contact with the practice for over two years and reactivate patients if they return to the practice at a later date
- Clean recall lists and outstanding action lists. Identify patients to be recalled and check BP records for recalled patients
- Update current medication list deleting old scripts, entering over-the-counter and complementary medicines, changing doses when relevant, flagging medications prescribed elsewhere and completing reason for prescribing
- Ensure diagnoses are updated and entered in the relevant field or removed if no longer applicable. Use maintenance program to convert old diagnosis text into a searchable diagnosis codes (history codes)
- Convert blood pressure readings into values in the relevant fields
- Run data extractions and save results at each extraction date
- Print data quality reports for practice and GPs
- Print patient lists for data quality reports
- Respond to data quality queries from NPS by clarifying missing or conflicting data and update records before next extraction date
